# Bidirectional ventricular tachycardia in a patient with pheochromocytoma: A case report

**DOI:** 10.1016/j.hrcr.2026.05.015

**Published:** 2026-06-02

**Authors:** Soesja G. Pinto, Stefan A.J. Timmer, Charlotte E.P. Siegers, Malou van den Boogaard, Christian van der Werf

**Affiliations:** 1 Heart Center, Department of Clinical and Experimental Cardiology, Amsterdam UMC location University of Amsterdam, Amsterdam, The Netherlands; 2 Amsterdam Cardiovascular Sciences, Cardiomyopathy and Arrhythmia, Amsterdam, The Netherlands; 3 Noordwest Hospitalgroup location Alkmaar, Alkmaar, The Netherlands

**Keywords:** Pheochromocytoma, Bidirectional ventricular tachycardia, Catecholamine-induced ventricular arrhythmias, Reversed Takotsubo cardiomyopathy, Coronary spasms

**Key Teaching Points**

• Bidirectional ventricular tachycardia should prompt evaluation for secondary causes beyond primary cardiac disease.
• Reversed Takotsubo cardiomyopathy with unexplained reduced left ventricular ejection fraction may be triggered by catecholamine-secreting tumors such as pheochromocytoma.
• Early recognition and treatment of catecholamine-driven cardiomyopathy can result in complete recovery of cardiac function after tumor removal.

## Introduction

Pheochromocytoma is a rare, catecholamine-secreting neuroendocrine tumor arising from chromaffin cells of the adrenal medulla.^1^ Clinical presentation of pheochromocytoma can vary greatly, but often includes hypertension, headache, palpitations, and diaphoresis.^2^ In some cases, cardiovascular complications can occur. These include myocarditis, acute coronary syndromes, cardiomyopathy, heart failure, arrhythmias, and even sudden cardiac arrest.^3^, ^4^, ^5^ The most frequently observed arrhythmias in patients with pheochromocytoma are sinus tachycardia and atrial fibrillation. The occurrence of ventricular tachycardia (VT) in this setting is uncommon.^3^ The presence of bidirectional VT, generally a very infrequently observed VT subtype, is exceptionally rare in patients with pheochromocytoma.^6^, ^7^, ^8^, ^9^ This case report describes a patient with pheochromocytoma who presented with different VT subtypes, including bidirectional VT, combined with catecholamine-induced myocarditis on cardiac magnetic resonance imaging (MRI).

## Case report

A 52-year-old man presented to the emergency department with episodes of palpitations, nausea, vomiting, and pain localized to the neck, chest, and abdomen. He reported similar palpitations during a previous hospitalization 1 month earlier for suspected myocardial infarction, which was eventually classified as myocardial infarction with nonobstructive coronary arteries. During that previous admission, an adrenal tumor was incidentally detected and a biochemical evaluation for pheochromocytoma was initiated. Besides this previous hospitalization, the patient had no other significant medical history.

At the current presentation, the patient reported experiencing episodes of severe abdominal and back pain with a recent onset. He was found to be hypertensive, with a blood pressure of 198/124 mm Hg. Physical examination revealed diffuse abdominal tenderness, predominantly in the right upper quadrant and epigastrium.

**Figure 1:**
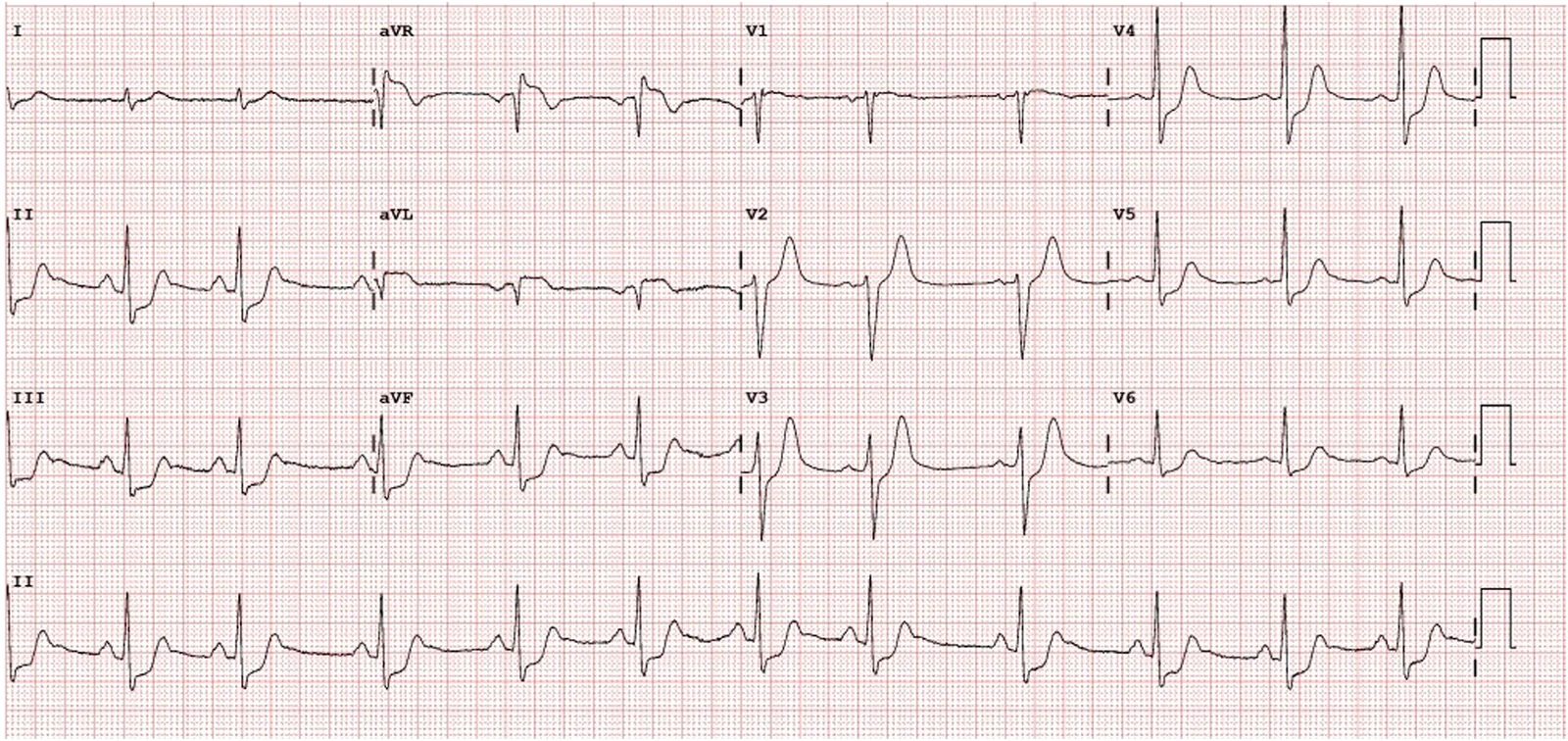
12-lead electrocardiogram showing initial electrocardiogram with diffuse ST-segment depressions and ST-segment elevation in leads aVR, aVL, and V1. Paper speed 25 mm/s; amplitude 10 mm/mV.

The initial electrocardiogram (ECG) demonstrated sinus rhythm at 70 beats/min with a narrow QRS complex, along with ST-segment elevation in leads aVR, aVL, and V1 and ST-segment depressions in the other leads, suggesting global subendocardial ischemia (Figure 1).

**Figure 2:**
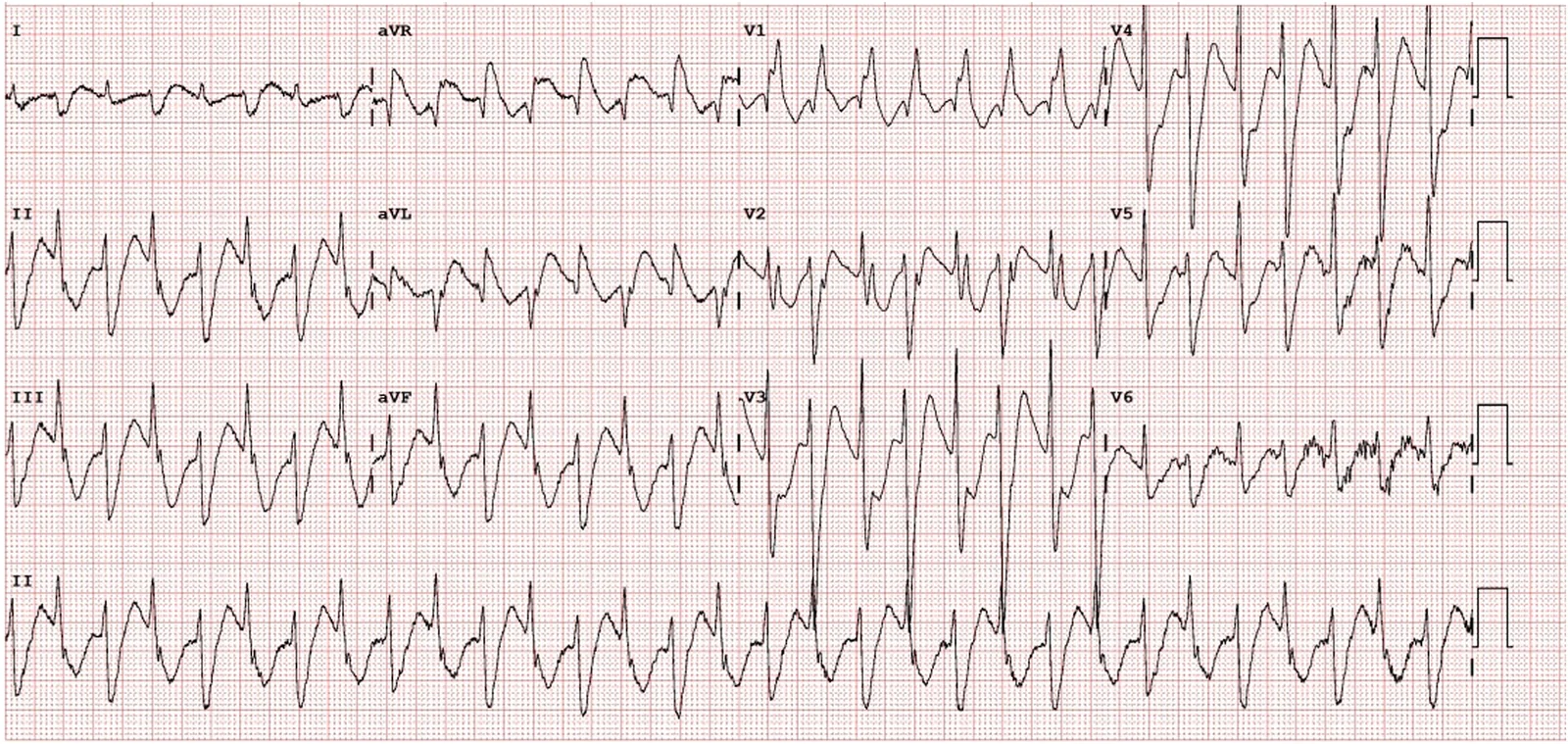
12-lead electrocardiogram showing bidirectional ventricular tachycardia with right bundle branch block morphology, alternating rightward and leftward axis, and cycle length of 320 ms. Paper speed 25 mm/s; amplitude 10 mm/mV.

**Figure 3:**
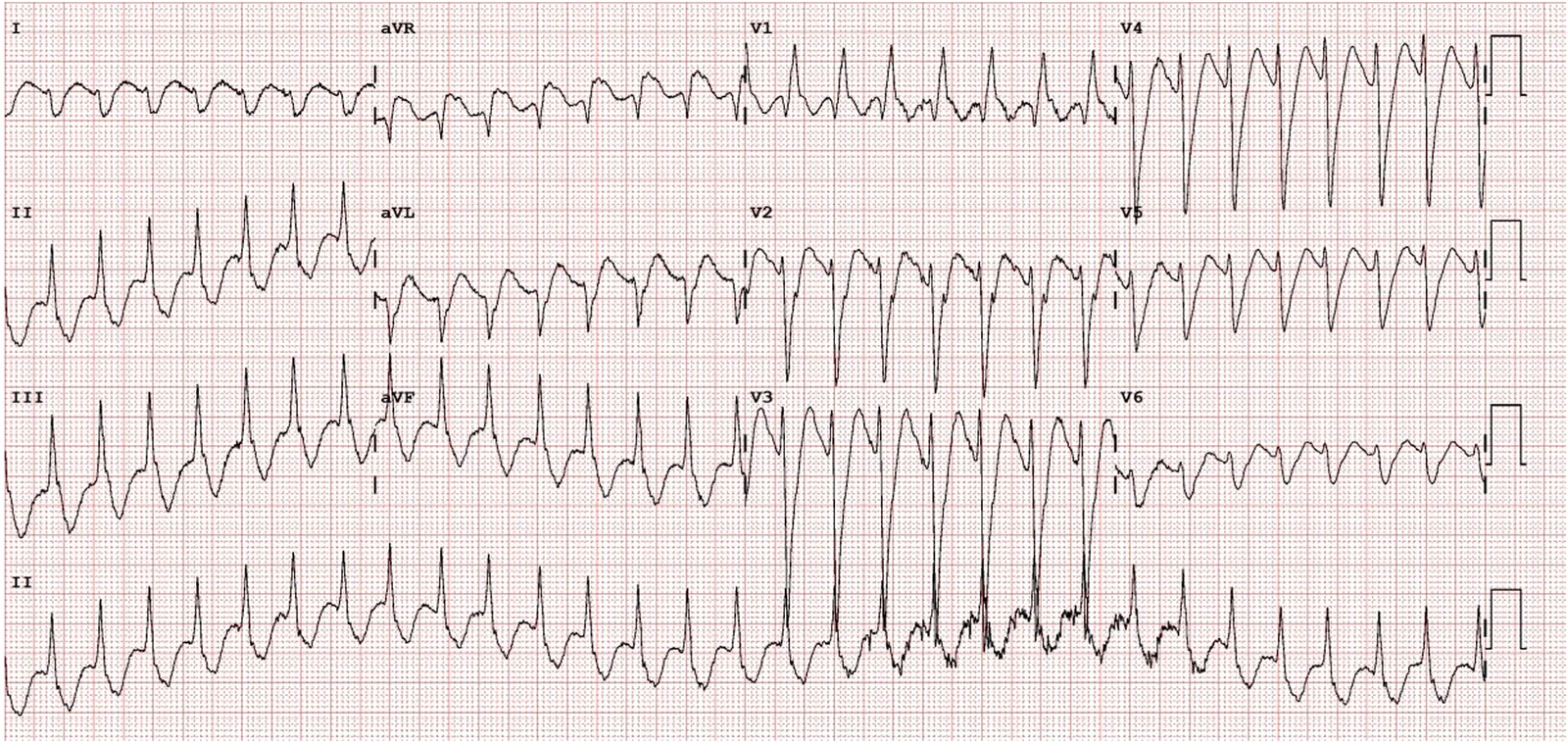
12-lead electrocardiogram showing monomorphic ventricular tachycardia with right bundle branch block morphology, rightward axis, and cycle length of 320 ms, most compatible with left anterior fascicular ventricular tachycardia. Paper speed 25 mm/s; amplitude 10 mm/mV.

Upon presentation, shortly after the patient experienced episodes of nausea and vomiting accompanied by sinus bradycardia, a bidirectional VT occurred (Figure 2), which subsequently transitioned to a monomorphic VT (Figure 3). Owing to this transition, a monomorphic wide complex tachycardia owing to supraventricular tachycardia with phase 3 aberrancy was considered less likely. In addition, the QRS morphology is most compatible with left anterior fascicular VT. During these episodes of VT, the patient was hypertensive, with a blood pressure of 177/107 mm Hg. The VTs were treated with a single dose of intravenous amiodarone and magnesium, resulting in conversion to sinus rhythm. Oral diltiazem was subsequently initiated to prevent suspected coronary spasms.

During hospitalization, the patient developed fever, dyspnea, and a productive cough. Together with an elevated B-type natriuretic peptide of 809 pmol/L (normal 0–15 pmol/L), these findings raised concern for either hospital-acquired pneumonia or acute decompensated heart failure. Antibiotics and diuretics were commenced. Chest radiograph displayed a typical batwing pattern and signs of interstitial pulmonary edema, consistent with acute heart failure. The patient improved clinically soon after starting treatment.

Laboratory testing showed an elevated troponin I of 770 ng/L (normal <20 ng/L), along with elevated liver enzymes (aspartate aminotransferase, alanine aminotransferase, lactate dehydrogenase, gamma-glutamyl transferase). In addition, the previously initiated plasma level evaluations of metanephrine (1.16 nmol/L; normal <0.040 nmol/L) and normetanephrine (5.40 nmol/L; normal <0.74 nmol/L) showed significant increases. Electrolyte disturbances were excluded. However, acid-base analysis and a urine drug screen were not performed, given that these investigations are not routinely obtained in the absence of clinical suspicion, which was not present in this case.

**Figure 4:**
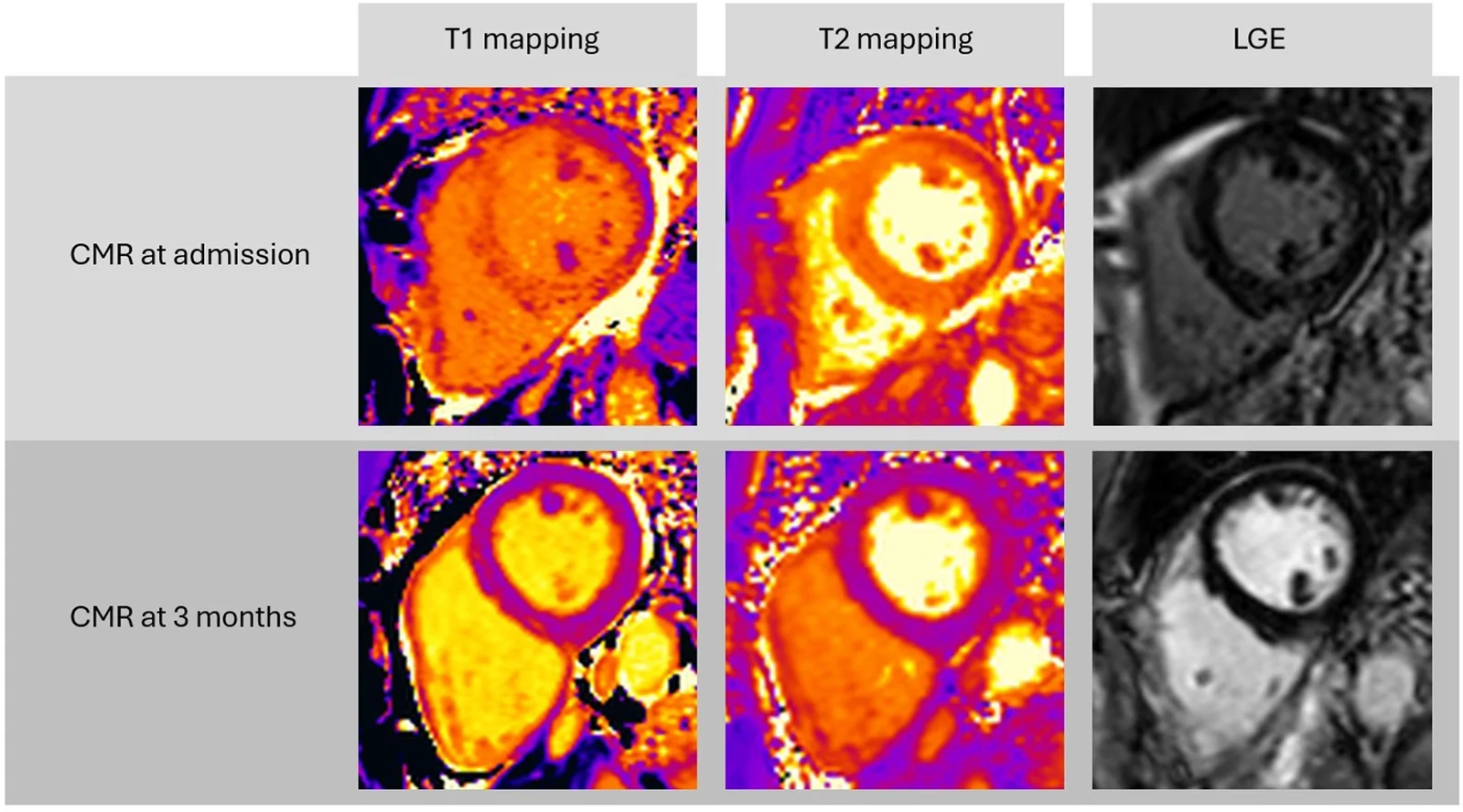
Cardiac magnetic resonance imaging at admission and at 3-month follow-up. T1 and T2 mapping. The initial scan shows abnormalities of the septum on both T1- and T2-weighted images. Late gadolinium enhancement is observed intramurally in the septal region and at the right ventricular insertion points. CMR = cardiac magnetic resonance; LGE = late gadolinium enhancement.

Transthoracic echocardiography revealed preserved left ventricular systolic function with new regional wall motion abnormalities (hypokinesia of the anteroseptal and basal anterior segments), normal right ventricular size and function, elevated pulmonary arterial pressures (45 + 15 mm Hg), and a dilated inferior vena cava with reduced respiratory variation. Cardiac MRI showed a dilated left ventricle with preserved left ventricular systolic function and elevated T1 values of 1470 ms, mid average 1210 ms, and apical average 1094 ms (normal 1000–1050 ms) and T2 values of 60 ms (normal 45–50 ms) (Table 1). Late gadolinium enhancement was observed in the intramural septal region and at the right ventricular insertion points. These findings were considered indicative of myocardial edema and inflammation and suggestive of either viral or catecholamine-induced myocarditis. In addition, cardiac MRI also revealed hypokinesia of the basal walls accompanied by apical hyperkinesia (Figure 4). This pattern could be suggestive of inverted Takotsubo cardiomyopathy.

**Table 1:** Comparison of left ventricular dimensions, volumes, and systolic function at admission and at 3-month follow-up

Parameter	Admission	3-mo follow-up
LVEDD	57 mm	49 mm
LVEDV	194 mL (107 mL/m^2^)	132 mL (65 mL/m^2^)
LVESV	91 mL (50 mL/m^2^)	51 mL (25 mL/m^2^)
LVSV	103 mL (57 mL/m^2^)	81 mL (40 mL/m^2^)
LVEF	53%	61%
IVSd	12 mm	9 mm
PWd	11 mm	7 mm

Values are presented as absolute measurements; indexed values (mL/m^2^) are corrected for body surface area.

IVSd = interventricular septal thickness at end-diastole; LVEDD = left ventricular end-diastolic diameter; LVEDV = left ventricular end-diastolic volume; LVEF = left ventricular ejection fraction; LVESV = left ventricular end-systolic volume; LVSV = left ventricular stroke volume; PWd = posterior wall thickness at end-diastole.

The combination of the clinical presentation, a history of an incidental adrenal lesion noted on previous imaging, and markedly elevated catecholamine levels strongly suggested the possibility of pheochromocytoma. Therefore, the patient was transferred from the referring hospital to a university hospital, where urgent adrenalectomy was performed and the diagnosis of pheochromocytoma was confirmed histopathologically. The postoperative course was uncomplicated, and no arrhythmia recurrences occurred.

Although an implantable cardioverter-defibrillator could have been considered, it was considered not indicated owing to the reversible cause of the VT, which was resolved with the adrenalectomy as treatment of the pheochromocytoma. Although there is a theoretical risk of recurrence of pheochromocytoma, the patient was postoperatively closely monitored, and no VT recurrences occurred. Therefore, a (wearable) defibrillator was not deemed necessary.

At the 3-month follow-up, the patient underwent repeat cardiac MRI, which demonstrated complete normalization of left ventricular morphology and function (Figure 4 and Table 1). Native T1 values and T2 values had returned to the normal range, and the previously observed reversed Takotsubo pattern was no longer present.

## Discussion

This case is particularly noteworthy owing to the occurrence of bidirectional VT in a patient with pheochromocytoma. Bidirectional VT is characterized by a 180-degree alternating QRS axis in the frontal plane. This VT is classically associated with catecholaminergic polymorphic VT, Andersen-Tawil syndrome, and digoxin intoxication. The underlying mechanism typically involves delayed afterdepolarizations triggered by calcium overload in the His-Purkinje system.^10^ In pheochromocytoma, excessive catecholamine release may produce a similar pathophysiological effect via beta-adrenergic overstimulation.

The cardiac imaging findings in this patient add a layer of interest to the case. The presence of basal hypokinesia with apical hyperkinesia is characteristic of inverted (reversed) Takotsubo cardiomyopathy. Although Takotsubo cardiomyopathy is a recognized cardiac manifestation of pheochromocytoma,^11^ most likely resulting from catecholamine excess, the inverted variant represents a much rarer subtype, with only a few cases reported in the literature.^12^^,^^13^

Finally, during the acute presentation, coronary vasospasm was considered as an additional diagnosis based on the observed ECG changes. However, several arguments in this case weigh against this diagnosis, including the fact that the ECG findings may be sufficiently explained by the severe myocarditis, and the absence of ventricular fibrillation argues against the degree of vasospasm that would have been required to produce this pattern. Therefore, the diagnosis of coronary vasospasms was never confirmed and remains uncertain.

The coexistence of these uncommon cardiac manifestations in a single patient illustrates the broad spectrum of cardiovascular complications that catecholamine excess in pheochromocytoma can produce.

## Conclusion

This case illustrates that pheochromocytoma should be considered in patients with unexplained VT, especially when accompanied by signs of catecholamine excess, including (reversed) Takotsubo cardiomyopathy, and evidence of vasospastic myocardial ischemia. Early recognition and definitive treatment through adrenalectomy can lead to complete resolution of symptoms and prevent potentially life-threatening complications.
